# Effects of Argon in the Acute Phase of Subarachnoid Hemorrhage in an Endovascular Perforation Model in Rats

**DOI:** 10.1007/s12028-024-02090-3

**Published:** 2024-08-22

**Authors:** Harald Krenzlin, Dominik M. A. Wesp, Anika A. E. Korinek, Henning Ubbens, Jakob Volland, Julia Masomi-Bornwasser, Katharina J. Weber, Dominik Mole, Clemens Sommer, Florian Ringel, Beat Alessandri, Naureen Keric

**Affiliations:** 1 Department of Neurosurgery, University Medical Center, Johannes Gutenberg University Mainz, Langenbeckstrasse 1, 55131 Mainz, Germany; 2 Institute of Neuropathology, University Medical Center, Johannes Gutenberg University Mainz, Mainz, Germany

**Keywords:** Argon, Subarachnoid hemorrhage, Neuroprotection, Endovascular perforation model

## Abstract

**Background:**

Subarachnoid hemorrhage (SAH) is a devastating disease with high morbidity and mortality. Neuroprotective effects of the noble gas argon have been shown in animal models of ischemia. The aim of this study was to investigate the effects of argon in the immediate early phase of SAH in a rat model.

**Methods:**

A total of 19 male Wistar rats were randomly assigned to three treatment groups. SAH was induced using a endovascular filament perforation model. Cerebral blood flow, mean arterial blood pressure (MAP), and body temperature were measured continuously. Group A received 2 h of ventilation by 50% argon/50% O_2_ (*n* = 7) immediately following SAH. Group B underwent a sham operation and was also ventilated by 50% argon/50% O_2_ (*n* = 6). Group C underwent SAH and 50% O_2_/50% N_2_ ventilation (*n* = 6). Preoperative and postoperative neurological and behavioral testing were performed. Histology and immunohistochemistry were used to evaluate the extent of brain injury and vasospasm.

**Results:**

The cerebral blood flow dropped in both treatment groups after SAH induction (SAH, 63.0 ± 11.6% of baseline; SAH + argon, 80.2 ± 8.2% of baseline). During SAH, MAP increased (135.2 ± 10.5%) compared with baseline values (85.8 ± 26.0 mm Hg) and normalized thereafter. MAP in both groups showed no significant differences (*p* = 0.3123). Immunohistochemical staining for neuronal nuclear antigen demonstrated a decrease of hippocampal immunoreactivity after SAH in the cornu ammonis region (CA) 1–3 compared with baseline hippocampal immunoreactivity (*p* = 0.0127). Animals in the argon-ventilated group showed less neuronal loss compared with untreated SAH animals (*p* < 0.0001). Ionized calcium-binding adaptor molecule 1 staining showed a decreased accumulation after SAH + argon (CA1, 2.57 ± 2.35%; CA2, 1.89 ± 1.89%; CA3, 2.19 ± 1.99%; DG, 2.6 ± 2.24%) compared with untreated SAH animals (CA1, 5.48 ± 2.39%; CA2, 4.85 ± 4.06%; CA3, 4.22 ± 3.01%; dentate gyrus (DG), 3.82 ± 3.23%; *p* = 0.0007). The neuroscore assessment revealed no treatment benefit after SAH compared with baseline (*p* = 0.385).

**Conclusion:**

In the present study, neuroprotective effects of argon occurred early after SAH. Because neurological deterioration was similar in the preadministration and absence of argon, it remains uncertain if neuroprotective effects translate in improved outcome over time.

## Introduction

Spontaneous aneurysmal subarachnoid hemorrhage (aSAH) is a devastating disease affecting often young and healthy patients [1]. The incidence of SAH is around 10 per 100,000 people [1, 2]. Despite continuous improvement on all aspects of care, case fatality within 28 days after SAH is around 50% [3, 4]. Long-term mortality is high, with standardized mortality ratios of 1.7 (95% confidence interval 1.4–2.1) overall and 3.2 (95% confidence interval 0.8–13.1) for patients under 40 years [4, 5]. A total of 8–20% of all patients remain severely disabled [6]. aSAH caused by a rupture of a cerebral aneurysm leads to a sudden increase of the intracranial pressure, short-term global ischemia, and disruption of the blood–brain barrier [7]. These changes during the first 72 h after the bleeding are subsumed as early brain injury (EBI) [8]. Those who survive the initial hemorrhage suffer from secondary complications, such as hydrocephalus, increased intracranial pressure, oxidative stress, vasospasm, and delayed cerebral ischemia [6, 9]. The extent of secondary damage defines mortality and morbidity and remains difficult to treat [10].

Primary treatment techniques include clipping, coiling, and neurocritical care [3]. Nimodipine, a calcium antagonist, is the only established neuroprotective agent in the treatment of aSAH [11–14]. The effectiveness is mostly empirical, and its mechanism of action not fully understood [12, 14]. By blocking calcium channels, nimodipine reduces Ca^2+^ influx in brain cells and prevent free radical formation [12]. Further, smooth muscle relaxation is thought to reduce the occurrence of vasospasm [12, 15]. The attention of research and treatment has shifted toward the pathophysiologic mechanisms during the first 72 h following bleeding to address EBI [16]. Early intervention strategies targeting EBI are an important approach for the development of novel neuroprotective therapies [17]. So far, there are no specific therapeutic options for the treatment of EBI [10]. Neuroprotective drugs have either failed to provide reproducible benefits in clinical trials or are still under investigation [18].

In recent years, the organoprotective and neuroprotective properties of noble gases have become a focus of medical research [12]. Despite their inability to undergo covalent bindings, various interactions of noble gases with their surroundings are reported [19, 20]. The electron configuration, lacking charge and polarity, is essential for chemical and biological effects of all noble gases [20]. It allows their passing through the blood–brain barrier and the interaction with distinct protein regions leading to structural changes [19]. Among all noble gases, the effects of xenon have been well demonstrated in vitro and in vivo [21, 22]. The effect of xenon on brain injury, neurological outcome, and survival after aSAH is currently investigated in clinical trials [23]. Because of high costs and the need of semiclose circuits in xenon treatment, alternative noble gases, such as helium, krypton, and argon, gained research attention [5, 19]. Argon is the most abundant without hypnotic effects. Promising neuroprotective effects of argon have been reported in different in vitro and in vivo models of ischemia and SAH [5, 19].

The aim of this study was to investigate the immediate early neuroprotective effect of argon postconditioning and neurological performance in the acute phase of SAH in a perforation-induced aSAH-model in rats.

## Material and Methods

### Study Design

To investigate the neuroprotective potential of argon after SAH 19 male Wistar rats (body weight 250–350 g; Charles River, Sulzfeld, Germany) were randomly assigned to three treatment groups. SAH was induced using the endovascular filament perforation model. Monitoring of intracranial pressure, CBF, and cerebral perfusion pressure was performed starting 10 min prior to SAH induction and continued for 60 min. Rats in group A received 2 h of ventilation by 50% argon/50% O_2_ (*n* = 7) immediately following SAH. Rats in group B underwent a sham operation and were also ventilated by argon and O_2_ (*n* = 6). Rats in group C underwent SAH and ventilation using or 50% O_2_/50% N_2_ (*n* = 6). After the procedure, all animals were then returned to their housing and were killed and subjected to histological workup after 3 days.

### Animal Care and Anesthesia

The study protocol and care procedures are in accordance with the guidelines of the German animal protection law (§6 Abs. 1 Satz 2 Nr. 4 TschG) and were approved by the local committee for animal welfare (Landesuntersuchungsamt Rheinland-Pfalz, approval no AZ 177-07/G 12-1-064). Species-appropriate housing was assured in macrolone cages in an animal room, with free access to food and water and 12-h day–night cycles. Anesthesia induction was achieved by isoflurane (1-Chloro-2,2,2-triflouroethyldiflouromethylether; Forene) 100% V/V by vaporization (DRÄGER Vapor 19.3) at 1013 mbar with 1 Vol% for 30 s. Sedation was maintained by subcutaneous application of weight adapted ketaminhydrochloride (Park-Davis GmbH Ketanest, 68 mg/kg bodyweight) and medetomidinhydrochloride (zoetis Dorbene vet, 0.36 mg/kg bodyweight). An arterial line (polyethylene catheter, outer diameter 0.96 mm, MAPB; Gould transducer) was inserted into the tail artery for blood pressure monitoring and arterial blood gas analyses (pO_2_, electrolytes, glucose, pH). Body temperature was kept to physiological level via feedback-controlled heating pad (Harvard Apparatus, MA).

### Surgical Procedure

**Fig. 1:**
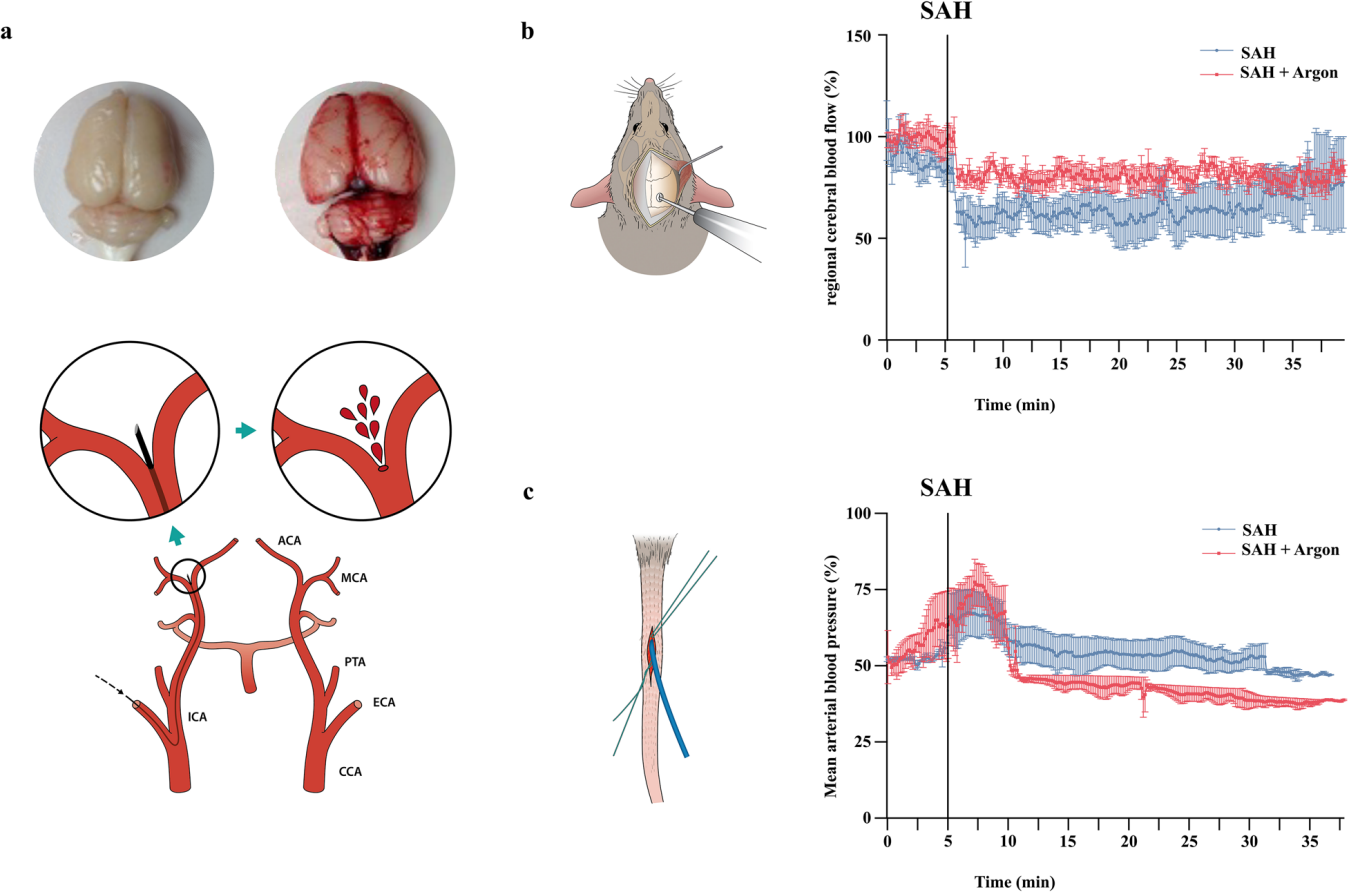
**a** Schematic presentation of vessel perforation model for SAH induction. **b** Presentation of regional cortical blood flow method and results. **c** Presentation of canulation of tail artery and invasive blood pressure monitoring results

Rats were fixed into a stereotactical frame during mircosurgical trephination (operation microscope; Zeiss, Wetzlar, Germany) was performed. A laser Doppler probe was placed 5 mm lateral and 1 mm occipital to the bregma (Model BPM 403a; Vasomedics, St. Paul, MN). The regional cortical blood flow was measured continuously to ensure appropriate cerebral perfusion. Regional cortical blood flow changes (laser Doppler) were related to baseline values assessed prior to surgical manipulation. The animals were then operated on in a supine position. A midline neck incision was performed to expose the left common carotid artery, internal carotid artery (ICA), and external carotid artery. A 4–0 monofilament nylon suture (PROLENE; Ethicon Johnson & Johnson Medical GmbH) sharpened at the tip was introduced into the ICA and advanced carefully. For artery perforation, the suture was advanced for 2 mm at the bifurcation of the ICA into the anterior cerebral artery and middle cerebral artery. Sham-operated animals had no vessel perforation (Fig. 1a).

### Treatment

Argon/oxygen ventilation was performed using a mixture of 50 vol% argon and 50 vol% (Fa. Linde Gas Therapeutics GmbH, Oberschließheim, Germany; 50% argon 5.0, oxygen 4.5). The gas mixtures were applied under normobaric conditions at 5 bar and room temperature of 21–24 °C using a rat face mask. Capnometry and blood pressure monitoring were performed continuously, while blood gas analyses were performed preoperatively, intraoperatively, and postoperatively. The gas mixture was administered for 60 min after the respective procedure. At the end of the treatment, the antidotes atimapezolhydrochloride (Pfizer Alzane, 2 mg/kg bodyweight) and tramadolhydrochloride (AbZ Tramadol, 2 mg/kg bodyweight) were administered intraperitoneally. For additional analgesic therapy, 1.25 ml tramadolhydrochloride (Tramdadol, 100 mg/ml), was added to the drinking water.

### Neurological Testing

A scoring system (neuroscore) was compiled to quantify sensory and motor integrity by evaluating motor activity, orientation, and reaction to tactile, visual, and auditory stimuli [24]. Testing was performed in a quiet room in dim light. Before operation, all animals were trained daily before the procedure*.* The points obtained in the individual tests were then summed to yield an overall score ranging from 0 points (no deficit) to 6 points (most severe deficit). Status assessment was performed before and 24 h after SAH. In addition, the righting reflex was examined. To assess the righting reflex, the rat is placed on its back on a tabletop and the time taken for the animal to right itself through 180° is measured.

### Histology

Twenty four hours after SAH, animals were transcardially perfused with 4% buffered paraformaldehyde, and their brains were carefully removed and postfixed for 24 h. Coronal section slices with a thickness of 5 µm spaced 250 µm apart were made through the paraffin-embedded brains and stained with hematoxylin and eosin to delineate the injury. The damaged area on each section was photographed with a charge coupled device, a specific type of camera (CCD) (SSC-C370P, Sony) connected to a light microscope (Axiopod 2; Zeiss, Oberkochen, Germany). The areas of ischemic brain damage at the traumatized hemisphere were surveyed with image analyzing software (Optimas 6.51, VSG, UK). Sections were immunohistologically stained for ionizing calcium-binding adaptor molecule 1 (Iba-1) (microglia and macrophages, 1:300; Wako Chemicals) and neuronal nuclear antigen (NeuN) (1:100; Santa Cruz Biotechnology, INC). Immunoreactive areas were calculated in a region of interest (size, 150 × 300 μm).

### Statistical Analysis

One-way analysis of variance was applied for comparative testing. All data were expressed as means ± standard deviations and 95% confidence intervals. Statistical analyses were performed using Graph Pad Prism 8 software. *P* < 0.05 was considered statistically significant.

## Results

### SAH Induction and Monitoring

Intraventricular hemorrhage was found in one animal (100%) in the SAH + argon group, not in those with normal ventilation. Intracerebral hemorrhage was present in three animals (25%) with SAH + argon ventilation and in one animal (16.7%) in those without argon ventilation.

Invasive mean arterial blood pressure (MAP) measurement was performed to assess argon induced changes. During SAH, MAP increased to 135.2 ± 10.5% of baseline and normalized thereafter. In the SAH group, MAP was 104.5 ± 8.2 mm Hg at baseline and increased to 136.0 ± 17.1 mm Hg during SAH. The MAP then normalized to 85.8 ± 26.0 mm Hg during the follow-up period. During wound closure and treatment MAP decreased to 97.7 ± 18.0 mm Hg and remained stable. MAP in both SAH groups showed no significant differences (*p* = 0.3123). No MAP changes were detected in sham-operated animals (Fig. 1b).

The CBF dropped in both SAH groups, reaching minimum values after SAH induction (63.0 ± 11.6% of baseline). In both SAH groups, initial CBF was 141.7 ± 71.7 laser doppler unit (LDU) at baseline and decreased to 127.4 ± 67.6 laser doppler unit (LDU) after SAH. During the observational period, the CBF recovered without fully reaching pre-SAH values. CBF decline and recovery during the observational period were statistically significant but less pronounced in animals under argon ventilation (*p* < 0.0001). No CBF changes were observed in sham-operated animals. All CBF changes were of statistical significance within each group compared with baseline values (*p* < 0.001; Fig. 1c).

### Vessel Morphology

**Fig. 2:**
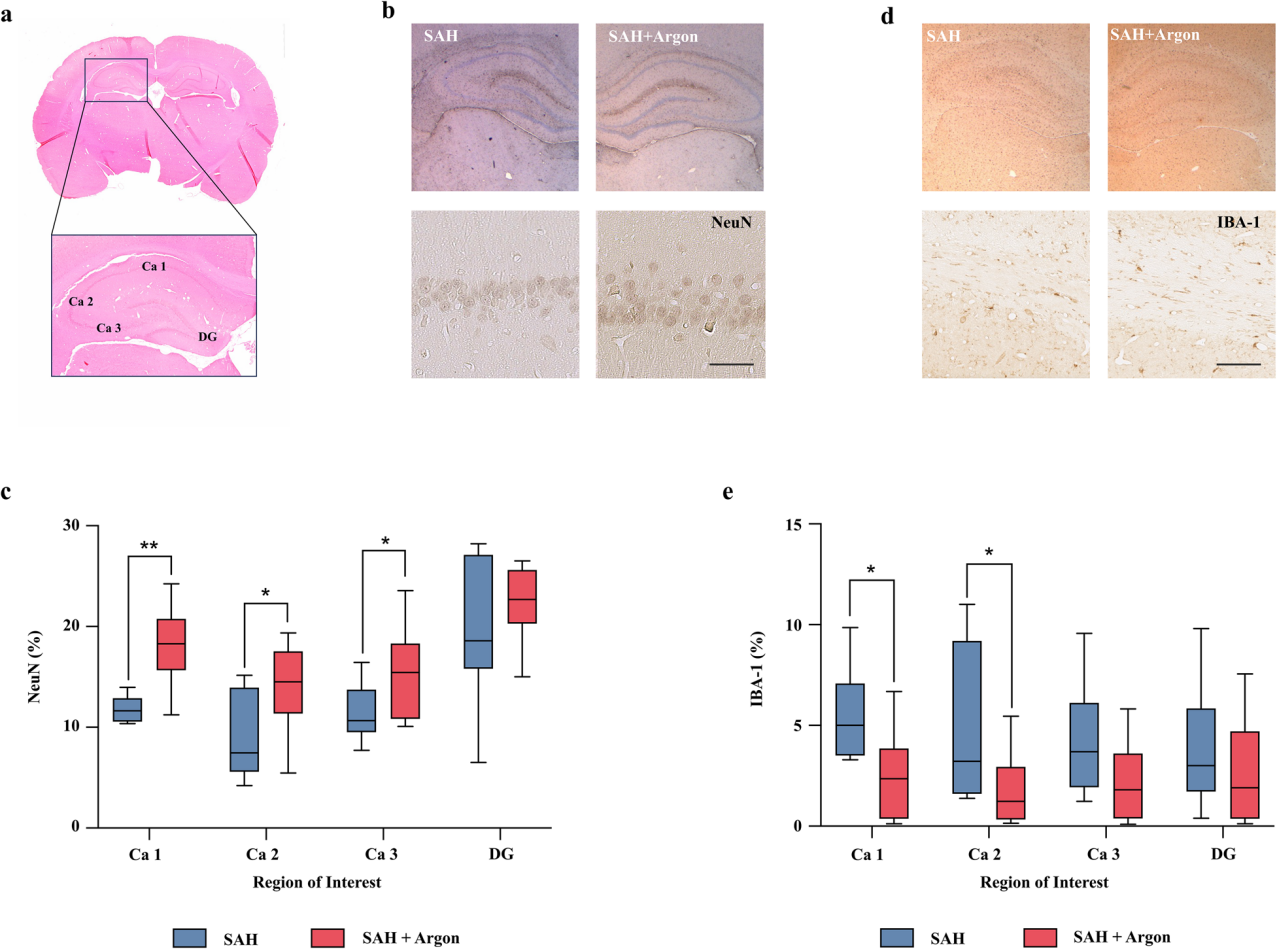
**a** HE-stained basilar artery for measurement of vessel diameter and vessel wall thickness. **b** Results of the measurements

Vasogenic effects of argon ventilation were assessed through analyses of vessel morphology. The vessel diameter of the basilar artery in sham-operated animals was 234.1 ± 38.0 µm. In those with SAH, basilary artery diameter was significantly increased to 296.5 ± 11.4 µm indicating vasodilation (*p* = 0.0096). In those animals with SAH + argon, vessel diameter was 195.3 ± 11.1 µm (*p* = 0.11). The examination of the wall thickness of the basilar artery showed no differences, with a wall thickness of 14.8 ± 2.7 µm (sham), 12.2 ± 3.1 µm (SAH), and 12.2 ± 3.3 µm (SAH + argon), respectively (*p* = 0.3434; Fig. 2).

### Hippocampal Neuronal Damage

**Fig. 3:**
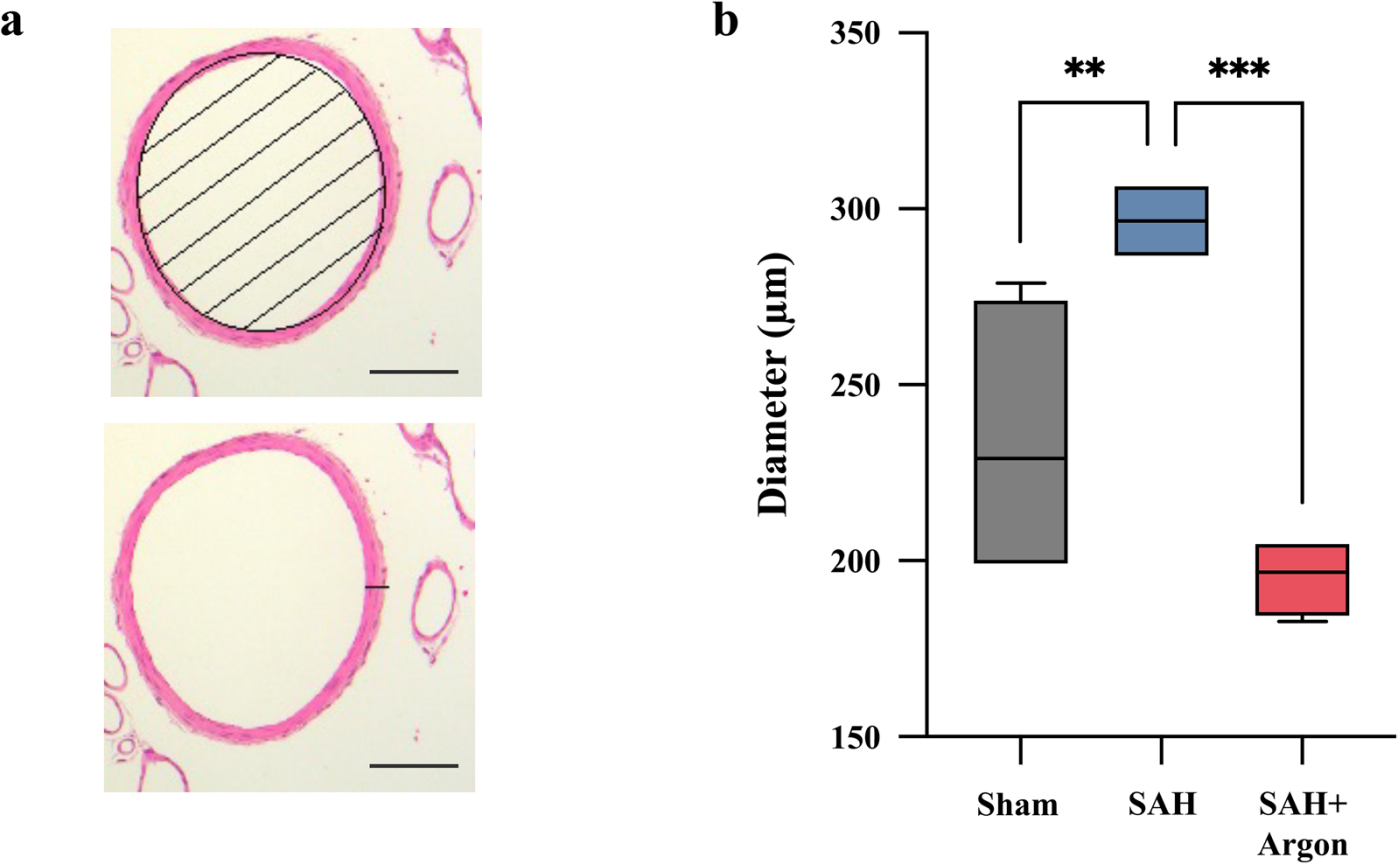
**a** HE-stained cross section of the rat brain depicting the analyzed regions. **b**, **d** NeuN-staining shows a significant decreased neuronal damage in argon-ventilated SAH group. **c**, **e** IBA-1 staining shows a significant decreased accumulation in argon-ventilated SAH group

The NeuN disappears from damaged or dying pyramidal neurons in the hippocampus due to hypoxia or brain injury [25]. NeuN is a common biomarker indicating neuronal damage starting 12 h after injury [26]. Immunohistochemical staining for NeuN demonstrated a decrease of hippocampal immunoreactivity after SAH in the cornu ammonis regions (CAs) 1–3 (CA1, 18.0 ± 3.7%; CA2, 14.2 ± 4.1%; CA3, 15.1 ± 4.2%; dentate gyrus (DG), 22.4 ± 3.6%) compared with untreated SAH animals (CA1, 11.9 ± 1.3%; CA2, 9.5 ± 4.6%; CA3, 11.4 ± 2.9%; DG, 19.7 ± 7.4%; *p* = 0.013). Animals in the argon-ventilated SAH group showed significantly less neuronal loss compared with untreated SAH animals (*p* < 0.0001). However, there were no differences between the argon-ventilated SAH animals and sham-operated animals (*p* = 0.57; Fig. 3a–c).

Iba-1 staining (% surface area) showed a decreased accumulation after SAH + argon (CA1, 2.57 ± 2.35%; CA2, 1.89 ± 1.89%; CA3, 2.19 ± 1.99%; DG, 2.6 ± 2.24%) compared with untreated SAH animals (CA1, 5.48 ± 2.39%; CA2, 4.85 ± 4.06%; CA3, 4.22 ± 3.01%; DG, 3.82 ± 3.23%) alone (*p* = 0.0007) (Fig. 3d and e).

### Neuroscoring

**Fig. 4:**
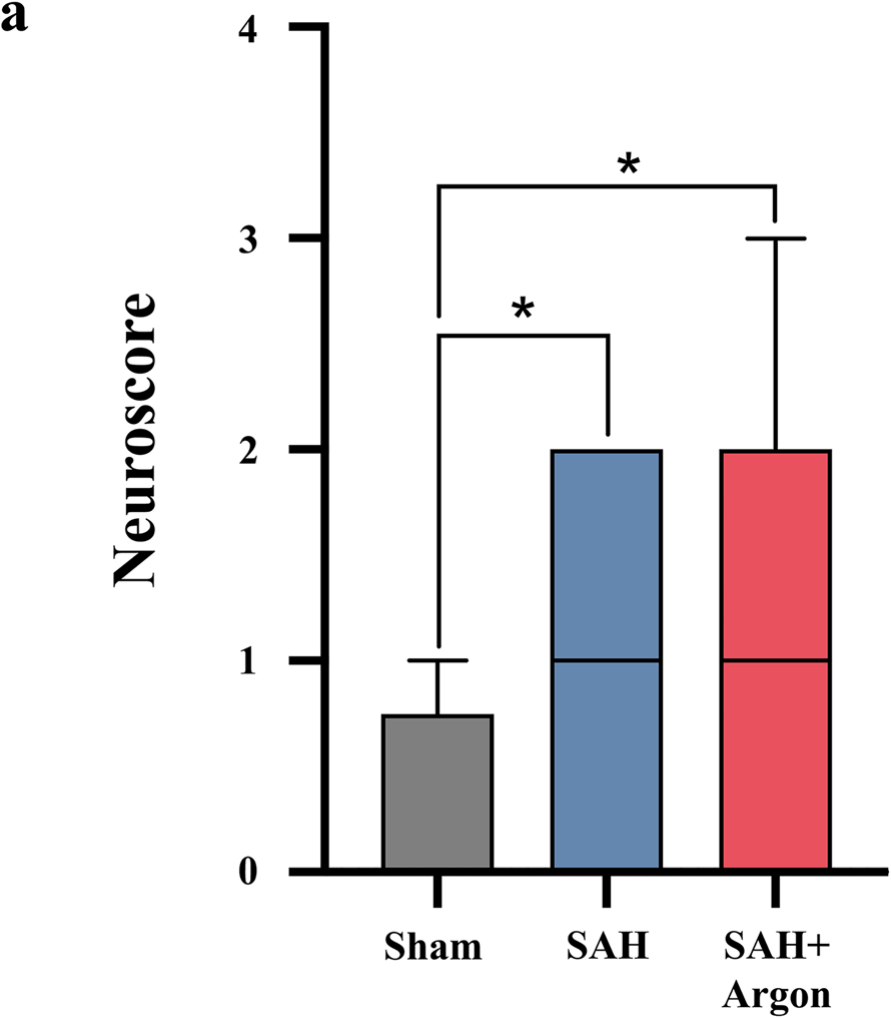
The postoperative neuroscore is similar among the treated aSAH groups

The neuroscore assessment was used to evaluate motor and behavioral deficits in the different treatment groups. All animals were without deficits prior to SAH. Twenty four hours after surgery, those with SAH were significantly worse compared with sham-operated animals (*p* = 0.0141). No differences were detected between animals with SAH and animals with SAH + argon (*p* = 0.385; Fig. 4).

## Discussion

Argon has long been associated with neuroprotection in vitro and in vivo [27]. Early studies indicated an improved resistance toward hypoxia under an argon atmosphere [19]. In vitro, different experimental models investigated the protective effects of argon under different hypoxic conditions. Among those, fetal organotypic murine brain slices subjected to trauma imitating traumatic brain injury and oxygen-glucose-deprivation are the most common [27, 28]. In vivo*,* argon’s neuroprotective and immunomodulatory properties were studied in endovascular perforation models of SAH [5].

The presented data add several novel findings to the increasing evident of the neuroprotective potential of argon in SAH. For the first time, continuous neuromonitoring has been applied in an endovascular perforation model of SAH in rats under argon postconditioning. MAP did not differ in rats receiving argon ventilation compared to those breathing ambient air. These results fall in line with previous studies where argon had no effect on MAP in a rat middle cerebral artery occlusion model and male humans under general anesthesia [29, 30]. Further, no difference in MAP was detected between argon treatment and controls in a controlled cortical impact model for traumatic brain injury in mice [27]. Likewise, argon ventilation after perinatal asphyxia in newborn piglets did not lead to changes of heart rate, blood pressure, cerebral saturation and electrocortical brain activity [31]. Furthermore, there were no changes to arterial blood gas analysis during the time of argon postconditioning. These findings validate the safeness of argon inhalation after SAH and extend previous observations of argon treatment in models of ischemia and traumatic brain injury [27, 30].

In a rat isolated lung model vasodilation was observed in animals with argon ventilation [32, 33]. To assure constant brain perfusion, cerebral blood vessels are equipped with different regulatory mechanisms subsumed as autoregulation [34]. An increase in blood pressure leads to vasoconstriction, a decrease in vessel diameter and a decrease in blood pressure leads to vasodilation [34, 35]. Attenuation of the consequences of increased CBF, such as blood–brain barrier disruption and vasogenic edema are predominantly mediated by vasoconstriction [34]. During cerebral ischemia, these mechanisms become dysfunctional, resulting in cerebrovascular dysregulation that cannot compensate for the reduction in CBF [36]. However, in models of cerebral ischemia in rats, no changes in MAP and CBF were observed under argon ventilation [30]. In the present data, a reduced vessel diameter in argon-ventilated animals could limit excessive CBF and ultimately contribute to neuroprotection. It is of note that despite a significant reduction in vessel diameter, no changes of global CBF are observed in this model. This observation falls in line with previous data in which argon did not lead to significant changes in cerebral circulation or metabolism not only in preclinical models but also in male patients under fentanyl-midazolam anesthesia [30].

In a dose-dependent manner an average of 50% argon diminished mechanical and metabolic stress after ischemia [37]. Argon postconditioning in a middle cerebral artery occlusion in rats lead to reduced infarct volumes and hippocampal damage in vivo [38]. Similar neuroprotective properties of argon and amelioration of microglial activation 72 h after SAH has been reported using a similar SAH model [5, 39]. Mechanisms of neuroprotection include an increase of B-cell lymphoma 2 (Bcl-2) that promotes cell survival, a change in signaling through extracellular signal-regulated kinase 1/2 in neurons and glial cells and an indirect effect mediated by the γ-aminobutyric acid receptor (GABAA) receptor [40–42]. The presented data indicate a protective effect of argon ventilation after experimental SAH on neuronal cell death and microglial activation pattern. Here, the nuclear protein NeuN disappears from pyramidal neurons in the hippocampus indicating dying and damaged cells. Loss of NeuN is attenuated under argon ventilation. This emphasizes previous data of argon-mediated injury reduction in organotypic hippocampal brain slices from mice subjected to oxygen–glucose deprivation [43]. However, 24 h after SAH statistically significant differences were observed only in the DG hippocampal region [39]. Here, in the very early stage 12 h after SAH, NeuN preservation is seen in all hippocampal areas including CA1–3 rather than the DG indicating a more generalized neuroprotective potential than previously expected.

Microglia contribute to ischemia-induced neuronal damage through proinflammatory cytotoxic cytokines and immunosuppression [44]. Microglia accumulation after SAH is linked to neuronal cell death in murine and human specimen [45]. Subsequently, a reduction of microglia significantly reduced neuronal cell death in these models [45]. Nevertheless, microglia polarization and their occurrence in active or resting states are thought to exert either detrimental or even beneficial effects [46]. Microglial activation after SAH increases over time [47]. This stands in contrast to a previously reported peak of the neuroprotective argon effect 24 h after SAH [39]. The data presented here show a neuroprotective effect of argon mediated through attenuated damage caused by excessive CBF and reduced accumulation of Iba-1 positive cells very early after SAH. Although Iba-1 is not solely restricted to microglia but also found on other cells of monocytic lineage, attenuate microglia accumulation through argon ventilation might contribute to the observed neuroprotective effects. It remains unclear if this effect persists over time or vanishes during the course of the disease. It is stipulated in literature that short treatment periods with argon (e.g., 1 h) may account for the short-term effect [19, 39]. Interactions of microglia with a wide range of different surrounding cells further increase the complexity of secondary damage formation after SAH [48]. Limited sustainability of the neuroprotective argon treatment effect might necessitate repeated treatment regiments to undo this shortcoming.

## Conclusions

Argon postconditioning after SAH does not lead to MAP changes while improving cerebral perfusion in the acute phase after bleeding. Vasodilation is prevented and neuronal damage is decreased in animals with argon ventilation predominant in the hippocampal areas CA1–3 12 h after SAH. Concordantly, Iba-1–positive cell accumulation is lessened, mitigating secondary neuroaxonal damage. However, decreases in neuronal damage does not translate into improved neurological performance status in the early stage after bleeding. It remains to be seen of repeated periods of argon ventilation might sustain the effects of neuroprotection and ultimately lead to improved long-term neurological outcome.

## Limitations

The results presented in this analysis are confined to a very early time point after SAH. The data show that neuroprotective effects of argon postconditioning after SAH are observed early after the bleeding but potentially vanish over time. Larger studies with more animals and time points are needed because the neuroprotective effect of argon is becoming more evident.
